# Identifying Infectious Agents in Snakes (Boidae and Pythonidae) with and Without Respiratory Disease

**DOI:** 10.3390/ani15152187

**Published:** 2025-07-25

**Authors:** Marline M. Faulhaber, Florence Tardy, Anne V. Gautier-Bouchardon, Sabine Öfner, Sebastiaan Theuns, Sieglinde Coppens, Elisabeth Müller, Michael Pees, Rachel E. Marschang

**Affiliations:** 1 LABOKLIN GmbH & Co.KG, Labor für klinische Diagnostik, Steubenstraße 4, D-97688 Bad Kissingen, Germany; marlinefaulhaber@bundeswehr.org (M.M.F.); mueller@laboklin.com (E.M.); 2 Anses, Ploufragan-Plouzané-Niort Laboratory, Mycoplasmology, Bacteriology and Antimicrobial Resistance Unit, F-22440 Ploufragan, France; florence.tardy@anses.fr (F.T.); anne.bouchardon@anses.fr (A.V.G.-B.); 3 Auffangstation für Reptilien München e.V., Kaulbachstraße 37, D-80539 München, Germany; sabine.oefner@reptilienauffangstation.de; 4 PathoSense, Poortakkerstraat 41A, B-9051 Gent, Belgium; sebastiaan.theuns@pathosense.com (S.T.); sieglinde.coppens@pathosense.com (S.C.); 5 Department of Small Mammal, Reptile and Avian Medicine and Surgery, University of Veterinary Medicine Hannover, Bünteweg 2, D-30559 Hannover, Germany; michael.pees@tiho-hannover.de

**Keywords:** snake, reptile, mycoplasma, respiratory disease, serpentovirus, python, boa

## Abstract

Mycoplasmas are known respiratory pathogens in tortoises, but few studies exist in snakes. To better understand the correlation with clinical signs and co-infections, samples from mycoplasma-positive snakes with and without clinical respiratory disease were analyzed. Oral swabs from 15 snakes (pythons *n* = 12, boas *n* = 3) were examined using polymerase chain reaction (PCR) and third-generation sequencing (TGS). Additionally, mycoplasma isolation assays were performed. Pathogens detected by PCR included Mycoplasmas (15/15, 100%), serpentoviruses (9/15, 60%), and *Chlamydia* sp. (2/15, 13%); those detected by TGS included Mycoplasmas (14/15, 93%), serpentoviruses (10/15, 67%), *Chlamydia* sp. (1/15, 7%), and 15 different bacterial species. Sequencing of the mycoplasma PCR products revealed a close genetic relationship to *Mycoplasmopsis agassizii*. TGS identified genetically distinct mycoplasmas and three different serpentoviruses. While mycoplasmas could not be successfully propagated, *Brucella intermedia* comb. nov. was identified in eight cultures. Respiratory disease in snakes is often multifactorial, involving various pathogens and environmental influences. This study demonstrates that comprehensive diagnostics are essential for understanding disease processes in snakes and improving the detection of diverse pathogens. Further research is needed to improve laboratory diagnostics for infectious diseases in reptiles and to better understand the roles of various pathogens in respiratory diseases in snakes.

## Simple Summary

Respiratory disease is common in snakes and often has multiple causes, including a plethora of infectious agents. Mycoplasmas are bacteria that can cause respiratory disease in tortoises, but not much is known about them in snakes. To learn more about how mycoplasmas relate to disease and other infections, we examined samples from 15 snakes, including boas and pythons, that were known to be mycoplasma-positive with and without respiratory disease, using two molecular methods, polymerase chain reaction, and third-generation sequencing, as well as culture methods for mycoplasma isolation. A number of pathogens were detected by each method, including mycoplasmas, serpentoviruses, *Chlamydia* spp., and other bacteria species. This study shows that using a full set of diagnostic tools can help understand multiple infections better and allow a more accurate detection and identification of different pathogens. More research is needed to improve laboratory testing for reptile diseases and to learn more about how these pathogens affect the respiratory system in snakes.

## 1. Introduction

Respiratory diseases are a common concern in the management of snake collections [1,2,3,4]. While bacterial pathogens are commonly implicated in respiratory infections in snakes [1,3,5,6,7], several viruses, including serpentoviruses, paramyxoviruses, reoviruses, adenoviruses, and sunviruses, have also been identified as important primary respiratory pathogens [8,9,10,11,12].

Viruses of the order *Nidovirales* contain linear, single-stranded RNA [13,14] and include notable human and animal pathogens [15,16,17]. Recently, nidoviruses detected in snakes have been classified within the subfamily *Serpentovirinae* (suborder *Tornidovirineae*, family *Tobaniviridae*) [14], together with nidoviruses found in other reptiles and in snake-associated nematodes [14,16]. These serpentoviruses, particularly those found in snakes, exhibit genetic diversity, with phylogenetic analyses revealing multiple divergent serpentovirus clades that have been classified into several different genera [17,18]. To date, the International Committee on Taxonomy of Viruses (ICTV) has recognized and classified seven serpentovirus genera and twelve subgenera ((ICTV) 2022 [14]), with most serpentoviruses identified in snakes belonging to the genus *Pregotovirus*. Initially recognized as a cause of respiratory disease in pythons [19,20,21], related viruses have also been found in disease outbreaks in other snake species, as well as in lizards and turtles, both in wild populations and in managed care [22,23,24]. To date, serpentoviruses have most often been described in ball pythons and pythons in the *Morelia* genus [2,25,26], but have also been found in snakes from other families [2,18,25]. Serpentoviruses in snakes are mostly associated with respiratory and systemic disease [2,16,26,27]. An experimental study confirmed a pathogenic relationship between infection with ball python nidovirus (BPNV-1) (genus *Pregotovirus*) and disease, with clinical signs in infected ball pythons including excessive mucous production in the oral cavity, mucinous inflammation of the upper respiratory tract (URT), and cranial esophagitis [12]. Recent studies have also shown that asymptomatic infections are not uncommon [2,25].

Bacteria of the genus *Mycoplasma*, commonly named mycoplasmas, are known to play a significant role in diseases affecting the upper respiratory tract of various reptiles. *M*. [*Mycoplasmopsis*] *agassizii* and *M. testudineum* [*Mycoplasmopsis testudinea*] are two species of mycoplasmas known to cause upper respiratory tract disease in chelonians [28,29,30]. In snakes, reports of mycoplasma infection are still limited, but several case reports have linked mycoplasmas with respiratory disease in pythons [1,31,32]. In addition to respiratory issues, mycoplasmas have also been associated with other clinical signs in infected snakes, including stomatitis, anorexia, and weight loss [32,33]. A recent study analyzed mycoplasmas from various snake species phylogenetically, assigning them to six clusters (A–F) [34]. The majority of the mycoplasmas detected in that study were related to *M.* [*Mycoplasmopsis*] *agassizii* or *M. testudineum* [*Mycoplasmopsis testudinea*]. However, mycoplasmas were also detected that were only distantly similar to these in numerous cases, including mycoplasmas most closely related to the avian *M. anserisalpingitidis* and *M*. [*Mycoplasmopsis*] *iners*, the lizard *M*. [*Mycoplasmopsis*] *iguanae*, and the equine *M*. [*Mycoplasmoides*] *fastidiosum*. All studies of mycoplasmas in snakes have been in managed collections, and there are currently no known monitoring reports from wild snakes.

A major revision of mycoplasma taxonomy has recently been proposed [35,36]. Although the revised taxonomy has been adopted by major biological databases such as NCBI Taxonomy [37] and the List of Prokaryotic Names with Standing in Nomenclature (LPSN) [38], it remains under critical scrutiny due to unresolved inconsistencies in the classification of certain species [39]. In this study, we therefore use the overarching designation *Mycoplasma* spp. (with the revised genus names indicated in parentheses) to ensure clarity and consistency as well as to make comparisons with previous studies on mycoplasmas in reptiles easier.

Multifactorial disease processes with the presence of a variety of potential pathogens have been hypothesized to play a significant role in respiratory disease processes. Even in cases in which a single pathogen is identified as the primary cause of disease, secondary pathogens may strongly influence the course of disease, e.g., bacterial secondary infections in snakes infected with serpentoviruses [2,12].

The wide range of host species involved and the complexity in understanding the roles of various infectious agents in reptiles make infectious disease diagnostics in these animals challenging. Numerous PCR-based methods have been employed for detecting specific pathogens, often chosen to detect a range of related pathogens. Examples include the detection of adenoviruses [40], snake serpentoviruses [25], herpesviruses [41,42,43], or mycoplasmas [34]. In recent years, the increasing use of advanced molecular techniques such as third-generation sequencing (TGS) in reptile medicine is offering new insights into the presence and diversity of pathogenic agents in these animals [24,44,45,46]. Nanopore sequencing may be helpful in detecting potential pathogens, especially when classical diagnostic methods fall short. However, in many cases, cultivation remains a valuable diagnostic tool, as it allows further characterization and a deeper understanding of bacterial species, including their growth patterns, resistance profiles, and pathogenic potential, as well as determining the environmental conditions required for their growth, which molecular methods alone cannot always reveal [47].

This study aimed to compare multiple diagnostic methods for the detection of pathogens in snakes from a mixed collection of a reptile rescue center in Munich. The snakes tested were known to be infected with mycoplasmas, and several had clinical signs of respiratory disease. The goal of the study was to use classical molecular detection methods and nanopore sequencing to detect possible relevant pathogens in these snakes, to compare the results of the methods used, and to correlate the detection of specific infectious agents with clinical signs. In addition, attempts were made to culture mycoplasmas to better characterize the mycoplasmas found in snakes.

## 2. Materials and Methods

Snakes from a reptile rescue center (Auffangstation für Reptilien München e.V., Munich, Germany) which had experienced an outbreak of (severe) respiratory disease in parts of its collection were initially screened for mycoplasma infections using two different conventional polymerase chain reaction (PCR) methods [34]. Most of the animals originated from private households and breeding facilities and had been confiscated for animal welfare reasons. In addition, one snake had been submitted to the rescue center after being found, with its origin remaining unknown.

Fifteen mycoplasma-positive snakes were chosen for additional testing. All snakes underwent a basic external clinical examination including evaluation of posture, breathing, skin, body condition, eyes, cloaca, and oral cavity. Four oral swabs (cotton swabs, Paul Boettger GmbH & Co. KG, Bodenmais, Germany) were collected directly one after the other from each for PCR and third-generation sequencing (TGS), and two swabs were collected for mycoplasma isolation attempts. Routine diagnostic testing for respiratory pathogens using PCR was carried out by a commercial veterinary laboratory (Laboklin GmbH & Co. KG, Bad Kissingen, Germany). TGS (Oxford Nanopore Technologies, Oxford, United Kingdom) was carried out by PathoSense laboratory (Faculty of Veterinary Medicine, Ghent University, Merelbeke, Belgium), and mycoplasma isolation attempts were carried out by ANSES laboratory (Mycoplasmology, Bacteriology and Antimicrobial Resistance Unit (MBA) of the Ploufragan-Plouzané-Niort Laboratory (Ploufragan, France).

The examined snakes included members of five different species: ball python (*Python regius*) (*n* = 9), Dumeril’s boa (*Acrantophis dumerili*) (*n* = 3), Angolan python (*Python anchietae*) (*n* = 1), reticulated python (*Malayopython reticulatus*) (*n* = 1), and Sumatra python (*Python curtus*) (*n* = 1) (Table 1 and Table 2, Supplementary Tables S1 and S2).

The snakes in the present study were all kept under adequate conditions at the reptile rescue center. They were kept in individual terrariums, except for the three Dumeril’s boas, which lived together in one terrarium. The clinical status of the individual animals was assessed by an experienced veterinarian specialized in reptile medicine (S.Ö.) on the day of sampling in early autumn. The animals were classified into three groups: 1. Pythons that were considered clinically healthy on the day of sampling (Group 1); 2. Pythons that showed clinical signs of respiratory disease (Group 2); 3. The Dumeril’s boas that were kept together in one terrarium (Group 3), one of which had signs of disease, while the other two did not.

### 2.1. DNA Extraction and PCR

For the oral swabs collected for PCR, nucleic acid was extracted within one day of sample collection, as described previously [34], using a commercially available kit (MagNA Pure 96 DNA and Viral NA Small Volume Kit, Roche Diagnostics, Mannheim, Germany) according to the manufacturer’s instructions. After processing, the final volumes of 100 µL of the purified DNA samples were stored at 4 °C for 1 week, during which initial PCR testing was carried out. The samples were then frozen at −18 °C for storage.

All samples were screened for the following pathogens by PCR following previously published protocols: adeno-, reptarena-, ferla-, and reoviruses, and *Chlamydia* sp. [9,40,48,49,50]. Serpentoviruses and mycoplasmas were each detected using two separate PCRs. Serpentovirus PCR1 was performed according to Blahak et al. 2020 [25], and Serpentovirus PCR2 according to Stenglein et al. 2014 [20]. Mycoplasma PCR1 and PCR2 were performed as described previously [34]. The mycoplasma PCR2 was performed with a modification in the temperature profile to increase specificity: 95 °C for 15 min, nine “touch down” cycles at 95 °C for 45 s, 64.5 °C for 45 s (–1 °C per cycle), and 72 °C for 90 s, followed by primer annealing and polymerization: 30 cycles at 95 °C for 45 s, 56.5 °C for 45 s, and 72 °C for 90 s, then a final extension step at 72 °C for 10 min. Total volume used in all PCRs was 20 µL including 5 µL of DNA. The expected sizes of the conventional PCR products were verified by capillary electrophoresis using a QIAxcel Advanced system (QiagenQIAGEN GmbH, Hilden, Germany). Qualitative (negative/positive) PCR assays (real-time PCRs) were performed using the LightCycler^®^ 96 System (Roche Diagnostics GmbH, Mannheim, Germany). Ct-values below 35 were considered positive. Each PCR run included a negative and a positive control as well as an extraction control in each sample to assess nucleic acid extraction efficiency and PCR inhibition (DNA or RNA Process Control Detection Kit, Roche Diagnostics GmbH, Mannheim, Germany).

### 2.2. Sequence Analysis

To verify the specificity of the products from conventional PCRs, Sanger sequencing was attempted with all products that included a band of the expected size. Briefly, PCR products were purified using a MinElute purification kit (Qiagen, Hilden 40724, Germany) according to the manufacturer’s instructions and sequenced using both primers from the original PCR reaction using a Big-Dye Terminator v3.1 cycle sequencing kit (Life Technologies, Bochum 44799, Germany). Results were analyzed with an ABI 3130 sequencer (Applied Biosystems, Thermo Fisher Scientific, 64293 Darmstadt, Germany). Sequences were edited using Geneious Prime^®^ 2025.0.2 [51]; primer sequences were removed and the forward and reverse strands were assembled into a consensus sequence.

Identification of mycoplasmas to species level was performed, as previously described [34], by comparing the sequences with known ones in the NCBI databases [37,52] using Basic Local Alignment Search Tool (BLAST) [53], and leveraging the leBIBIQBPP tool [54].

### 2.3. Third-Generation Nanopore Metagenomic Sequencing

The freshly collected swabs were directly submitted to PathoSense (Ghent, Belgium). Transport was conducted using polystyrene boxes that were cooled with ice packs. Further preparation of the samples for viral and bacterial metagenomics and third-generation nanopore metagenomic sequencing was conducted using previously established and published protocols which rely on a proprietary process for collecting samples and identification of both viruses and bacteria [55,56,57,58]. All nucleotide sequences were classified using an in-house pipeline against the complete NCBI nucleotide (nt) database [37,52]. A spike-in control virus was introduced to each sample prior to the filtration step to ensure quality control and semi-quantification in downstream data analysis. The results reported by PathoSense were presented in semi-quantitative categories: very low, low, medium, high, and very high [57].

### 2.4. Mycoplasma Isolation and Matrix-Assisted Desorption/Ionization-Time of Flight Mass Spectrometry (MALDI-TOF)

Two swabs per snake from 11 of the 15 snakes examined (ball python (*n* = 7), Dumeril’s boa (*n* = 3), Angolan python (*n* = 1)) were collected for mycoplasma isolation assays (Supplementary Tables S1 and S2). All swabs were placed in 2.5 mL of buffered peptone water to obtain initial suspensions. Mycoplasmas were directly cultured by diluting 100 μL of initial suspension from each sample in 900 μL of Frey Medium 4 (FM4; [59], Arginine [60] or Friis [61] broth supplemented with antimicrobials (Amphotericin B (Sigma-Aldrich, Saint Quentin Fallavier, France) 25 μg/mL, Ampicillin (Sigma-Aldrich, Saint Quentin Fallavier, France) 2 units/mL, and Colistin (Sigma-Aldrich, Saint Quentin Fallavier, France) 75 µg/mL), and serial dilutions up to 10^−3^ were performed. All dilutions were incubated at 37 ± 2 °C or 30 ± 2 °C until the culture developed an acid color change or for a maximum of 30 days. When a color change occurred, or every 15 days, cultures were plated and incubated (37 ± 2 °C, 5% CO_2_) on Friis [61] or Indicia (Indicia biotechnologies, St Genis l’Argentière, France) agar medium and observed daily under a stereomicroscope to check if mycoplasma colonies could be observed.

Broth cultures showing a color change were analyzed by matrix-assisted desorption/ionization-time of flight mass spectrometry (MALDI-TOF, Bruker Daltonics, Bremen, Germany) as previously described [62]. Spectra were generated using a Microflex LT Biotyper operating system (Daltonics GmgH, Bremen, Germany). The data was analyzed by the Bruker Biotyper 3.0 software and the Bruker taxonomy library as well as a customized library for mycoplasmas.

## 3. Results

### 3.1. Clinical Signs

**Figure 1:**
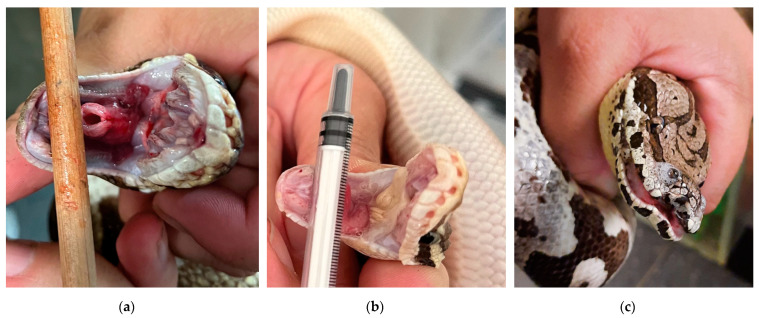
Clinical signs observed in individual snakes at the time of sample collection. (**a**) Ball python (*Python regius*) exhibiting mucous discharge, hyperemia of the mucous membranes, and mouth wheezing. (**b**) Ball python (*Python regius*) presenting with mucous discharge and a cream-colored coating on the oral mucosa. (**c**) Dumeril’s boa (*Acrantophis dumerili*) with mucous discharge and hyperemic mucous membranes.

Of the 12 pythons examined, 7 were classified as clinically healthy (Group 1), displaying no clinical signs (Table 1), while 5 exhibited respiratory signs (Group 2) (Table 2). The diseased snakes showed respiratory signs including mucous fluid or diphtheroid cream-colored membranes within the oral cavity, hyperemia of the mucous membranes, and wheezing (Figure 1). Audible respiratory sounds (wheezing) were observed in only two snakes. In the other animals, respiratory signs were present but not accompanied by any noticeable breathing sounds (Tabel 2). The pythons with respiratory signs were monitored closely. In the event of clinical worsening, lung secretions were collected via bronchoalveolar lavage. Antibiotic treatment with broad-spectrum antibiotics was initiated if abnormalities were detected, but in the pythons included in this study, the secretions were unremarkable.

Of the three Dumeril’s boas (Group 3) that were kept together, one displayed respiratory signs including nasal discharge, large amounts of mucous fluid in the oral cavity, hyperemia of the oral mucous membranes, and wheezing, while the two conspecifics appeared clinically healthy (Table 1 and Table 2, Figure 1). In the clinically affected boa, antibiotic treatment was initiated with ceftazidime (20 mg/kg s.c.) and ursocycline (initially 10 mg/kg s.c., then 5 mg/kg s.c.). After bacteriological culture and susceptibility testing of the lung lavage sample, therapy was switched to sulfamethoxazole/trimethoprim (15 mg/kg s.c.) for three weeks. Subsequently, a clinical improvement was observed.

Two of the examined snakes, a ball python and an Angolan python, died several weeks after the day of sample collection. One of these had previously shown no signs of clinical disease, while the other exhibited mucous fluid accumulation in the oral cavity on the day of sampling (Table 1 and Table 2, Supplementary Tables S1 and S2). Of the remaining snakes, the three boas have since died: one which had previously shown no signs of clinical disease in 2024, and the other two in 2025; of the latter two, one died peracutely and the other following euthanasia due to acute respiratory signs after experiencing recurrent respiratory issues.

### 3.2. Detected Pathogens

#### 3.2.1. Overview

Using PCR, mycoplasmas were detected in all 15 snakes (100%) using both PCR1 and PCR2, as expected, as previous detection of mycoplasmas was an inclusion criterion for the study. Serpentoviruses were detected with both PCRs in nine (9/15, 60%) and *Chlamydia* sp. were detected by PCR in two (2/15, 13%) of the examined snakes. No adeno-, reptarena-, ferla-, or reoviruses were detected by PCR in any of the animals tested (Table 3, Supplementary Tables S1 and S2).

Using TGS, mycoplasmas were detected in 14 (14/15, 93%), serpentoviruses were detected in 10 (10/15, 67%) and *Chlamydia* sp. in 1 (1/15, 7%) of the examined snakes. A variety of other, potentially pathogenic bacteria were also detected in various snakes (Table 3, Supplementary Tables S1 and S2).

#### 3.2.2. Identification of Mycoplasmas

PCR1 and PCR2 both detected mycoplasmas in all 15 snakes. However, Sanger sequencing only resulted in sequences that could be further analyzed in 12 cases for mycoplasma PCR1 and in 10 cases for mycoplasma PCR2 (Supplementary Table S3). The remaining PCR products generated sequences that, due to insufficient length or quality, could not be further evaluated. The sequence quality was sufficient for BLAST [53] analysis for at least one PCR product (from PCR1 or 2) from each snake. According to BLAST [53] analyses (Supplementary Table S3), all of the mycoplasmas detected (PCR1 and PCR2) showed nucleotide similarities ranging from 94% to 99% with mycoplasmas previously described in pythons and grouped in Cluster A [32,34,63]. LeBIBI analyses of the mycoplasma products of PCR1 also showed that the sequences shared high nucleotide similarity (ranging from 94% to 99%) to *M.* [*Mycoplasmopsis*] *agassizii* and *M. testudineum* [*Mycoplasmopsis testudinea*], corresponding to previously described sequences of snake mycoplasma clusters A and B (GenBank accession numbers: MZ686534.1, MZ686541.1, MZ686543.1, KU862617.1, U09786, AY366210) [34] (Supplementary Table S3). Using TGS, mycoplasmas identified as *M.* [*Mycoplasmopsis*] *agassizii*, *M*. [*Mycoplasmoides*] *fastidiosum*, *M*. [*Mycoplasmopsis*] *iguanae*, *M*. [*Mycoplasmopsis*] *pulmonis, M*. *testudineum* [*Mycoplasmopsis testudinea*], *Mycoplasmopsis* sp., and *Mesomycoplasma* sp. were detected in various snakes. The amount reported ranged from very low to high (Table 3, Supplementary Tables S1 and S2).

Infection with multiple *Mycoplasma* spp. occurred in 93% of the snakes sampled (*n* = 14/15) when considering both diagnostic methods together. Multiple *Mycoplasma* spp. were detected in all of the snake samples for which Sanger sequencing failed for either PCR1 or PCR2. However, multiple *Mycoplasma* species were also detected using TGS and PCR in samples in which Sanger sequencing was successful (Supplementary Tables S1–S3).

#### 3.2.3. Mycoplasma Cultures and MALDI-TOF

**Figure 2:**
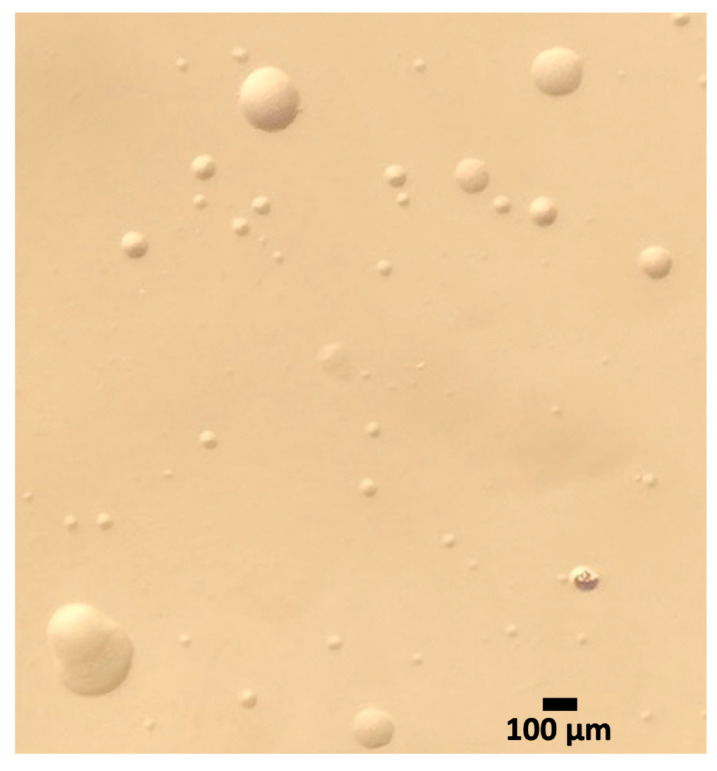
Example of mycoplasma-like colonies observed under a stereomicroscope (×120) after 12 days of incubation at 37 ± 2 °C and 5% CO_2_ on Friis agar medium of broth cultures having shown a color change. Culture from a sample taken from a ball python (*Python regius*; K9601).

Many contaminations by fast-growing bacteria were observed in broth and agar cultures. *Brucella intermedia* comb. nov. (basionym: *Ochrobactrum intermedium*) [64] was identified in cultures from seven snakes (Table 3, Supplementary Tables S1 and S2). Several colonies with mycoplasma-like aspect were observed on Friis and Indicia agar media (Figure 2, Supplementary Tables S1 and S2), but attempts to further propagate these colonies in broth medium were unsuccessful.

#### 3.2.4. Identification of Serpentoviruses

Serpentoviruses were detected in nine snake samples (60%, *n* = 9/15) using both PCR1 and PCR2. Sequencing of PCR1 products was unsuccessful. PCR2 is a probe-based real-time PCR and the products were not sequenced. TGS was able to identify serpentoviruses in ten snakes, and the detected serpentoviruses were differentiated into three different species (Table 3, Supplementary Tables S1, S2 and S4). The amounts reported ranged from low to high. A Bellinger River virus-related virus was found in all three of the Dumeril’s boas included in the study. BLASTN analysis showed query coverage between 52% and 64% and up to 72% similarity with Bellinger River virus (GenBank NC_046956) for all three sequences (Supplementary Table S4). Carpet python nidovirus 1 was detected in six of the twelve pythons tested (50%, *n* = 6/12) including in five ball pythons and one Angolan python. BLASTN analysis showed query coverage between 94% and 100% and up to 97% similarity with carpet python nido virus 1 (GenBank MK722366.1 and MK722375.1) (Supplementary Table S4). A third serpentovirus was detected in one Sumatran python. BLASTN showed a query coverage of 98% and a percent identity of 84.5% to a *Serpentovirinae* sp. isolate (GenBank OR131603.1) from a blood python (*Python brongersmai*), but could not be further categorized to species level (Supplementary Table S4). The sequences obtained from the serpentoviruses by TGS have been submitted to GenBank (accession numbers PV975819 to PV975839). Comparison of serpentovirus detection by PCR and TGS showed that all nine animals that tested positive for serpentoviruses by PCR were also identified as serpentovirus-positive by TGS, including all snakes in which the Bellinger River virus-like virus and the carpet python nidovirus 1-like virus were detected. The one Sumatra python in which a different serpentovirus was detected by TGS was negative for serpentoviruses using both PCRs (Table 3, Supplementary Tables S1 and S2).

#### 3.2.5. Detection of Chlamydia sp.

Using PCR, a *Chlamydia* sp. was detected in two Dumeril’s boas (13%, *n* = 2/15). TGS detected a very low amount of *Chlamydia* sp. in one of the two PCR-positive boas (7%, *n* = 1/15). Due to insufficient data from assembled contigs, a reliable nucleotide similarity could not be calculated. Classification was performed based on individual reads; thus, an exact similarity value cannot be provided. The top BLAST hit was CP014639.1 (*Candidatus Chlamydia sanziniae*, strain 2742-308, chromosome) (Table 3, Supplementary Tables S1 and S2).

### 3.3. Clinical Signs and Pathogens

Multiple potential pathogens were found in each of the snakes examined, including both the diseased and the clinically healthy snakes (Table 1, Table 2 and Table 3, Supplementary Tables S1 and S2). Serpentoviruses were detected in 43% (*n* = 3/7) of the snakes in Group 1 (pythons that were considered healthy) and in 80% (*n* = 4/5) of the snakes in Group 2 (diseased pythons). The serpentovirus that could not be further differentiated at the species level was found in a clinically healthy Sumatran python. A comparison of the mycoplasma species found in the different groups by TGS showed that *M*. [*Mycoplasmoides*] *fastidiosum* was more frequently detected in diseased pythons (40%, *n* = 2/5) than in clinically healthy pythons (29%, *n* = 2/7). *M*. [*Mycoplasmopsis*] *iguanae* (20%, *n* = 1/5) was found exclusively in a diseased python, while *M*. [*Mycoplasmopsis*] *pulmonis* was only found in clinically healthy pythons (43%, *n* = 3/7) (Table 3, Supplementary Tables S1 and S2). Mycoplasmas with up to 99% nucleotide similarity to *M.* [*Mycoplasmopsis*] *agassizii* were found in all examined snakes by PCR (100%, *n* = 15/15), and in six snakes by TGS (40%, *n* = 6/15). Multiple mycoplasma species were detected in a total of eleven animals: seven in Group 1 (100%, *n* = 7/7), and four in Group 2 (80%, *n* = 4/5) (Table 3, Supplementary Tables S1 and S2).

*Escherichia* spp. was found more often in clinically healthy pythons (57%, *n* = 4/7) than in diseased pythons (20%, *n* = 1/5). *Brucella intermedia* was detected more often in diseased pythons (80%, *n* = 4/5) than in clinically healthy ones (29%, *n* = 2/7). *Chryseobacterium* spp. was detected slightly more often in diseased pythons (40%, *n* = 2/5) than in clinically healthy pythons (14%, *n* = 1/7). *Elizabethkingia* spp. was detected at similar rates in both clinically healthy (57%, *n* = 4/7) and diseased pythons (60%, *n* = 3/5) and *Flavobacterium* spp. was also found equally as often in clinically healthy (14%, *n* = 1/7) and diseased (20%, *n* = 1/5) pythons. Both *Pseudomonas* spp. (43%, *n* = 3/7) and *Citrobacter* spp. (14%, *n* = 1/7) were found only in clinically healthy pythons, while *Lysobacter pythonis* was detected in a single diseased python (20%, *n* = 1/5) (Table 3, Supplementary Tables S1 and S2).

Bellinger River virus-like virus was detected in all three Dumeril’s boas in Group 3, although only one showed signs of disease. Mycoplasmas related to *M.* [*Mycoplasmopsis*] *agassizii* were also found in all three boas by PCR, while NGS revealed the presence of *M*. [*Mycoplasmopsis*] *pulmonis* and *M*. [*Mycoplasmoides*] *fastidiousum* in all three. Both clinically healthy boas tested positive for *Chlamydia* sp., and one clinically healthy boa also had *Lysobacter pythonis*. On the other hand, *Bacteroides fragilis*, *Brucella intermedia*, *Providencia rettgeri*, and *Stutzerimonas stutzeri* were found exclusively in the boa with respiratory signs, while *Chryseobacterium* sp. and *Paracoccus* sp. were detected in both a clinically healthy boa and the boa exhibiting respiratory signs (Table 1, Table 2 and Table 3, Supplementary Tables S1 and S2).

The semi-quantitative pathogen load determined via TGS was markedly higher in clinically healthy snakes compared to those exhibiting clinical signs, encompassing both bacterial and viral agents (Supplementary Tables S1 and S2).

## 4. Discussion

In captivity, snakes are often affected by respiratory diseases, which are linked to a variety of infectious agents, including viruses, bacteria, fungi, and parasites [1,4,65,66]. In many infections, multiple pathogens can play a role in the development of disease, which complicates diagnosis [12,26,66]. This study employed PCR and TGS methods to identify potential multiple pathogens in snakes infected with mycoplasmas and to correlate pathogen detection with clinical signs. The results of PCR and TGS were largely congruent for the presence or absence of those pathogens detected using both methods (mycoplasma, serpentoviruses, *Chlamydia* sp.).

### 4.1. Prevalence and Characteristics of Mycoplasma Infections

Mycoplasmas closely related to previously described mycoplasmas in pythons [32,63] were found in all snakes. These mycoplasma species are closely related to *M.* [*Mycoplasmopsis*] *agassizii* and *M. testudineum* [*Mycoplasmopsis testudinea*], which are known to cause upper respiratory tract disease in tortoises [29,67,68,69]. In single reports, *M. agassizii*- and *M. testudineum*-like mycoplasmas have also been associated with respiratory diseases in snakes, including stomatitis, general respiratory signs, and pneumonia [31,32,33]. Previous studies also showed a frequent detection rate of similar mycoplasmas in snakes, although no information on the clinical condition of infected snakes was provided [34,63]. The presence of mycoplasmas in both symptomatic and asymptomatic snakes suggests a potential chronic or latent colonization, but the exact role of these bacteria in disease remains unclear. Interestingly, using TGS, genetically more distant mycoplasma species were also detected, so that several of the snakes tested were colonized by multiple mycoplasmas. Mycoplasmas similar to all of those detected here have been previously detected in snakes of different families [34]. However, that study did not report on possible associations between mycoplasma detection and clinical disease.

Further investigations (e.g., histology, cultivation) are necessary to better characterize and understand the different mycoplasma species in snakes, as well as to investigate whether individual mycoplasmas can play a role in diseases and whether they can act as primary pathogens. The various attempts to isolate mycoplasma strains in different culture media and at different incubation temperatures failed to isolate any strains due to a high load of other contaminating bacteria. It is well known that a variety of bacterial species, including commensals, are commonly found in the oral cavities of snakes, which may contribute to these contamination issues [1,5,70,71,72]. However, mycoplasma-like colonies were observed on agar media. The isolation attempts were carried out using oral swabs. However, a previous study showed that mycoplasmas could be isolated from deeper tissues, such as tracheal or pulmonary tissue samples, but not from respiratory tract secretions [31]. It would, therefore, be interesting to repeat isolation assays with tracheal swabs (on live snakes) or on deeper tissues collected during necropsies of snakes that have died due to respiratory problems.

### 4.2. Detection and Diversity of Serpentoviruses in Snakes

Serpentoviruses are known causes of severe respiratory disease in snakes [12,20,27] and have been found to cause persistent infections and inapparent infections in some cases [2]. In the present study, serpentoviruses were detected in both clinically ill and healthy snakes but were more commonly detected in the diseased pythons (80%) compared to healthy ones (43%). The serpentoviruses of snakes are known to be genetically diverse [18], and viruses in several different species and genera in the subfamily *Serpentovirinae* have been described in both pythons and boas [2,18,27]. This can make diagnosing serpentovirus infections challenging [17]. In the snakes examined here, PCR was able to detect serpentoviruses in samples from nine animals, while TGS detected serpentoviruses in ten animals. Identification of the detected viruses indicated that all of the viruses detected by PCR belonged to the genus *Pregotovirus*, including strains identified by TGS as carpet python nidovirus 1 (in the species *Pregotovirus moreliae*) [73] and strains identified as Bellinger River virus-like (species *Pregotovirus myuchelyis*). An additional serpentovirus identified in a single Sumatran python could not be further categorized to species level but shows similarity to a *Serpentovirinae* sp. isolate obtained from an oral swab from a blood python [74].

Carpet python nidovirus 1 was first described in 2020 in two euthanized carpet pythons with respiratory distress [27]. A third snake from the same clutch previously died due to severe pneumonia. Upon examination of the surviving snakes, which showed only mild mucus accumulation in the oral cavity, necropsy revealed serpentovirus-associated lesions in their upper airways, indicating an early stage of the disease [27]. In all 30 snakes examined in that study, respiratory signs ranged from mild mucus secretion from the oral and nasal cavity to severe acute, recurrent, or chronic dyspnea. This is largely congruent with the observed signs in four infected snakes (three ball pythons and one Angolan python), which showed mucous fluid and coating in the oral cavity, as well as wheezing. All serpentovirus-infected pythons that showed clinical signs also harbored genetically diverse mycoplasmas as well as multiple bacterial species. All of the non-mycoplasma bacterial species found in these five pythons have been previously found in the oral cavities of both healthy and diseased snakes [1,5,70,71,72,75,76,77].

Bellinger River virus was first described in a disease outbreak in Bellinger River snapping turtles (*Myuchelys georgesi*) in Australia, leading to a severe population decline [23]. The origin of the virus was not identified, and studies of sympatric reptiles have not yet demonstrated the presence of this virus in squamate reptiles in Australia [78]. The detection of a closely related virus in Dumeril’s boas in Europe was, therefore, unexpected. The virus was detected in all three of the Dumeril’s boas, although only one was clinically ill at the time of sampling. Nevertheless, it seems likely that the serpentovirus was involved in the disease observed in the ill boa. All three Dumeril’s boas also harbored the same mycoplasma species, including *M*. [*Mycoplasmopsis*] *agassizii, M. testudineum* [*Mycoplasmopsis testudinea*], *M.* [*Mycoplasmoides*] *fastidiousum*, and *M.* [*Mycoplasmopsis*] *pulmonis*-like mycoplasmas. Three other bacteria were exclusively found in the diseased boa, but not in the two co-housed but healthy boas: *Providencia rettgeri*, *Bacteroides fragilis*, and *Stutzerimonas stutzeri*, but all three have previously been found in healthy snakes [5,71,72,79].

### 4.3. Chlamydia spp. and Other Bacteria Species Identified

*Chlamydia* sp., most closely related to *Candidatus Chlamydia sanziniae,* was detected in the two Dumeril’s boas without respiratory signs. *Candidatus Chlamydia sanziniae* was described in a study that identified new *Chlamydia* species in healthy snakes [80,81]. *Chlamydia pneumoniae*, originally known as a human pathogen causing acute respiratory diseases, has been described in a wide range of other hosts, including other mammals, marsupials, amphibians, and reptiles [82]. In snakes, this is the most commonly detected *Chlamydia* species [83,84,85], although multiple genetically diverse *Chlamydia* spp. have also been described [81,86]. *Chlamydia* spp. infections are associated with a wide range of clinical signs including regurgitation, stomatitis, pneumonia, and general respiratory signs [83,87,88,89], but can also be found in clinically healthy snakes [81,86,90].

Both TGS and culture led to the detection of multiple bacterial species in individual snakes. The most commonly identified bacterial contaminant found during attempts to culture mycoplasmas was the Gram-negative bacterium, *Brucella intermedia* comb. nov. (basionym: *Ochrobactrum intermedium*) [64], which was detected in seven snakes; five of them presented clinical signs on the day of sampling. This bacterium is considered an opportunistic pathogen in humans and animals [91,92,93]. Reports of *Brucella intermedia* in reptiles are scarce; however, its detection in a tracheal sample of a boa with pronounced respiratory signs and histologically confirmed multifactorial disease, including bronchopneumonia, and other systemic findings [92], indicated a potential pathogenic role in snakes.

Several studies have observed the presence of pathogenic bacteria (e.g., *Chlamydia* spp., *Mycoplasma* spp., *Salmonella* spp., and *Pseudomonas* spp.) in snakes that tested positive for viral infections [1,63,94,95]. Particularly, mycoplasmas have been described in connection with serpentovirus infections [63,94]. A study in 2021 [63] investigated the relationships between the occurrence of serpentoviruses, mycoplasmas, and *Chlamydia* spp. in snakes originating from veterinary practices or zoological institutions. That study found that mycoplasmas were detected in 78.5% of serpentovirus-positive samples and in all chlamydia-positive samples, highlighting the potential role of co-infections in respiratory disease dynamics in snakes. It is, therefore, diagnostically relevant to screen snakes with respiratory disease for multiple infectious agents.

The semi-quantitative pathogen load determined via TGS appeared generally higher in clinically healthy snakes compared to those showing clinical signs of disease. This observation may indicate that a higher pathogen burden does not necessarily correlate with the presence of clinical disease and could reflect asymptomatic colonization. The detection of a higher load of potential pathogens in clinically healthy animals could also be influenced by the known presence of various commensal bacterial species in the oral cavity of snakes [1,5,70,71,72], which are frequently reported in this anatomical region.

### 4.4. Influence of Husbandry and Stress

Deficiencies in husbandry conditions, poor nutrition, or a compromised immune status can also influence disease progression or contribute to disease development [1,12,66,96,97]. Opportunistic commensals may cause disease in malnourished, poorly maintained snakes living under stressful conditions [1,5,98,99]. A study in 2010 showed a significant correlation between deficits in husbandry conditions and the presence of microorganisms and notable findings in snakes [65].

The snakes in the present study were all kept under adequate conditions at a reptile rescue center. However, movement of animals, which is commonly necessary at such a facility, can cause stress and may influence the immune status of animals as well as possibly exposing them to new potential pathogens.

### 4.5. Treatment

Although specific treatment protocols for mycoplasma infections in snakes are not yet well established, general therapeutic approaches are available for managing respiratory disease of suspected bacterial origin [3,66,100,101]. Rapid detection and intensive supportive care are often necessary. Affected snakes should be kept at the upper end of their preferred optimal temperature range, with appropriate humidity levels to support immune function. Fluid therapy is frequently required, particularly in dehydrated individuals [101]. Once the animal has been adequately rehydrated, antibiotic therapy may be initiated.

To minimize the risk of antimicrobial resistance, the use of broad-spectrum antibiotics should be avoided. Antibiotic selection should ideally be based on culture and sensitivity testing, such as disc diffusion or minimum inhibitory concentration (MIC) results [3,101]. Pharmacokinetic studies indicate that clarithromycin and oxytetracycline are preferred over fluoroquinolones for treating mycoplasmosis in reptiles [101]. The Dumeril’s boa was initially treated with a cephalosporin (ceftazidime) and oxytetracycline (ursocycline). Ceftazidime is considered a reserve antibiotic and its use should be justified—such as in this case, where it was chosen for its efficacy against gram-negative bacteria commonly involved in respiratory infections in snakes, its good tolerance, and its convenient dosing interval (every 72 h) [102,103]. Recent studies have shown that other antibiotics—including enrofloxacin (87.2%), marbofloxacin (89.4%), and trimethoprim/sulfamethoxazole (85.1%)—may be effective against gram-negative pathogens isolated from reptiles, supporting their targeted use in confirmed infections [104]. Oxytetracycline was selected due to its potential efficacy against *Mycoplasma* spp., as tetracyclines are considered a first-line treatment for mycoplasmosis in reptiles [66]. Following culture and susceptibility testing of a lung lavage sample, treatment was switched to sulfamethoxazole/trimethoprim.

It should be noted that evaluation of treatment was beyond the scope of the present study, and expanding diagnostic testing may not always make choosing specific treatments easier.

### 4.6. Challenges in Interpreting Bacterial Findings in Reptile Diagnostics

A common concern with NGS-based diagnostics is the large volume of data, which may obscure clinically relevant information [105]. The diverse microbial flora found in the examined snakes complicates the interpretation of results regarding the presence of potentially pathogenic organisms. Twelve different bacteria species were detected in all examined snakes, which have been previously described in healthy and in diseased snakes [1,5,70,71,72,75,76,77]. Understanding which of the detected bacteria are clinically relevant and worthy of treatment in a given animal can be challenging, regardless of the methodology used for bacterial detection. Previous studies have come to various conclusions regarding the use of numbers and quantity of specific bacteria in helping to identify clinically significant bacterial species in snakes with respiratory disease [70,106]. The finding of similar numbers of different bacteria in diseased (1–8 species identified) and clinically healthy (1–6 species identified) snakes and higher numbers of individual bacterial species in clinically healthy than in diseased snakes by TGS in the present study (Table 1, Supplementary Tables S1 and S2) supports previous conclusions regarding the difficulty of interpreting bacterial detection in snakes based on presence/absence or quantification data alone. Therefore, interpreting the clinical significance of certain bacterial findings requires careful consideration of the clinical context, and, ideally, correlation with additional diagnostic methods or clinical signs.

### 4.7. Limitations

The clinical evaluation of the snakes included in this study was based on observational data, but did not include auscultation or diagnostic imaging. Snakes can often hide signs of disease, and may not show visible signs of respiratory disease until a large portion of the respiratory tract is affected [1,107]. It is, therefore, possible that some of the snakes considered clinically healthy in the study were indeed suffering from some degree of respiratory or other disease that was not detected. A further limitation of this study was that the clinical examination was only conducted at the time of sample collection. More frequent examinations combined with pathogen identification would provide greater clarity regarding the progression of disease in relation to specific pathogens. Furthermore, it should be noted that four swabs were taken from each animal. To minimize potential variations in quality, the swabs were collected consecutively from the oral cavity. The oral swabs were transported on ice as quickly as possible to each of the three laboratories involved in the study. Oral swabs were considered an appropriate sample material [34,108], but tracheal washes could have provided more information on viruses and bacteria present in the lower respiratory tract.

## 5. Conclusions

This study provides a preliminary insight into mycoplasma infections in snakes in association with clinical signs and co-infections. Multiple infectious agents were detected in all of the snakes examined, both with and without respiratory signs, including multiple mycoplasmas and serpentoviruses. The findings suggest that mycoplasmas, in combination with serpentoviruses, may contribute to clinical disease, though the relevance of genetically distinct *Mycoplasma* species requires further investigation. Additionally, the role of mycoplasmas as primary infectious agents in snakes remains unclear. Cultivating mycoplasmas is essential for deeper characterization, and ongoing cultivation experiments will aid in understanding their role in infection and disease in snakes. This study also highlights the uses of both PCR and TGS for detecting pathogens in snakes, with TGS providing a rapid overview of all pathogens, including genetically diverse representatives of known species, and detecting co-infections with related pathogens. It enables single-molecule sequencing and real-time sequencing capture. In contrast, PCR offers higher sensitivity for individual pathogens. However, PCR methods also offer increased specificity, which can lead to negative test results in the case of infections with related but distinct organisms, as demonstrated by novel mycoplasmas and serpentoviruses missed by the PCR assays in this study. These findings should serve as a basis for further investigations to explore the relationship between mycoplasma presence and clinical signs in snakes.

## Figures and Tables

**Table 1:** List of the examined snakes that were considered clinically healthy at the time of sampling.

Pathogens	Detection Method	*Python regius* (Ball Python) K09608	*Python regius* (Ball Python) K09602	*Python regius* (Ball Python) K09609	*Python regius* (Ball Python) K02321	*Python regius* (Ball Python) K02325	*Python curtus* (Sumatra Python) K02330	*Malayo-* *python reticulatus* (Reticulated Python) K02324	*Acrantophis dumerili* (Dumeril’s Boa) K09605	*Acrantophis dumerili* (Dumeril’s Boa) K09603
Bellinger River-related virus	TGS	−	−	−	−	−	−	−	+	+
Carpet python nidovirus 1	TGS	+	−	+	−	−	−	−	−	−
Serpentovirus	TGS	−	−	−	−	−	+	−	−	−
Serpentovirus (PCR1, PCR2)	PCR	+	−	+	−	−	−	−	+	+
*Mycoplasma* *agassizii* like (PCR1, PCR2)	PCR	+	+	+	+	+	+	+	+	+
*M. [Mycoplasmopsis] iguanae*	TGS	−	−	−	−	−	−	−	−	−
*M. [Mycoplasmoides] fastidiosum*	TGS	−	+	−	−	−	+	−	+	+
*M. [Mycoplasmopsis] agassizii*	TGS	−	−	+	−	+	−	+	−	−
*M. [Mycoplasmopsis] pulmonis*	TGS	−	−	+	−	+	+	−	+	+
*Mycoplasmopsis* sp.	TGS	+	−	−	−	−	−	−	−	−
*M. testudineum* *[Mycoplasmopsis testudinea]*	TGS	+	−	−	−	−	−	+	−	−
*Mesomycoplasma* sp.	TGS	−	−	−	+	−	−	−	−	−
*Chlamydia* sp.	PCR	−	−	−	−	−	−	−	+	+
*Chlamydia* sp.	TGS	−	−	−	−	−	−	−	+	−
*Bacteroides fragilis*	TGS	−	−	−	−	−	−	−	−	−
*Brucella intermedia* comb. nov	MALDI- TOF	+	−	+	nd	nd	nd	nd	−	−
*Chryseobacterium* sp.	TGS	+	−	−	−	−	−	−	+	−
*Citrobacter* sp.	TGS	+	−	−	−	−	−	−	−	−
*Elizabethkingia* sp.	TGS	−	−	−	+	+	+	+	−	−
*Escherichia* sp.	TGS	−	−	−	+	+	+	+	−	−
*Flavobacterium* sp.	TGS	+	−	−	−	−	−	−	−	−
*Lysobacter* *pythonis*	TGS	−	−	−	−	−	−	−	+	−
*Paracoccus* sp.	TGS	−	−	−	−	−	−	−	+	−
*Providencia rettgeri*	TGS	−	−	−	−	−	−	−	−	−
*Pseudomonas* sp.	TGS	−	−	−	+	+	−	+	−	−
**Clinical signs**		None Deceased: 41 days after sampling (23 October 2023)	None	None	None	None	None	None	None Deceased 1 year, 6 months, and 19 days after sampling (31 May 2025)	None Deceased 1 year, 3 months after sampling (December 2024)

nd: not done. No samples for mycoplasma culture were collected from these animals. + = pathogen detected, − = pathogen not detected.

**Table 2:** List of the examined snakes that showed signs of disease at the time of sampling.

Pathogens	Detection Method	*Acrantophis dumerili* (Dumeril’s Boa) K09604	*Python* *anchietae* (Angolan Python) K09606	*Python regius* (Ball Python) K09601	*Python regius* (Ball Python) K09607	*Python regius* (Ball Python) K09610	*Python regius* (Ball Python) K09611
Bellinger River-related virus	TGS	+	−	−	−	−	−
Carpet python nidovirus 1	TGS	−	+	−	+	+	+
Serpentovirus	TGS	−	−	−	−	−	−
Serpentovirus (PCR1, PCR2)	PCR	+	+	−	+	+	+
*Mycoplasma agassizii* like (PCR1, PCR2)	PCR	+	+	+	+	+	+
*M. [Mycoplasmopsis] iguanae*	TGS	−	+	−	−	−	−
*M. [Mycoplasmoides] fastidiosum*	TGS	+	−	+	−	−	+
*M. [Mycoplasmopsis] agassizii*	TGS	−	+	−	−	+	+
*M. [Mycoplasmopsis] pulmonis*	TGS	+	−	−	−	−	−
*Mycoplasmopsis* sp.	TGS	−	−	+	−	−	−
*M. testudineum* *[Mycoplasmopsis testudinea]*	TGS	−	−	+	−	+	+
*Mesomycoplasma* sp.	TGS	−	−	−	−	−	−
*Chlamydia* sp.	PCR	−	−	−	−	−	−
*Chlamydia* sp.	TGS	−	−	−	−	−	−
*Bacteroides fragilis*	TGS	+	−	−	−	−	−
*Brucella intermedia* comb. nov	MALDI- TOF	+	+	+	+	−	+
*Chryseobacterium* sp.	TGS	+	+	+	−	−	−
*Citrobacter* sp.	TGS	−	−	−	−	−	−
*Elizabethkingia* sp.	TGS	−	+	−	+	−	+
*Escherichia* sp.	TGS	−	−	−	−	−	+
*Flavobacterium* sp.	TGS	−	−	−	−	−	+
*Lysobacter pythonis*	TGS	−	−	−	−	−	+
*Paracoccus* sp.	TGS	+	−	−	−	−	−
*Providencia rettgeri*	TGS	+	−	−	−	−	−
*Pseudomonas* sp.	TGS	−	−	−	−	−	−
**Clinical signs**		Nares: nasal discharge. Oral cavity: mucous fluid, hyperemia of the mucous membranes. Deceased 1 year, 6 months, and 12 days after sampling (24 May 2025).	Oral cavity: mucous fluid. Deceased: 13 days after sampling (25 September 2023).	Oral cavity: mucous fluid, hyperemia of the mucous membranes, wheezing.	Oral cavity: mucous fluid, cream-colored coating.	Oral cavity: mucous fluid wheezing.	Oral cavity: mucous fluid.

+ = pathogen detected, − = pathogen not detected.

**Table 3:** A detailed overview of the pathogens detected, stratified by diagnostic approach and their frequency of occurrence among the analyzed snake specimens.

Pathogens	Detection Method	No. of Positive Snakes/ All Examined Snakes (%)	Diseased Pythons: No. Positive/All Diseased Pythons (%)	Healthy Pythons: No. Positive/ All Healthy Pythons (%)	Boas: No. Positive/ All Boas * (%)
**Viruses**					
Bellinger River-related virus	TGS	3/15 (20%)	0/5 (0%)	0/7 (0%)	3/3 (100%)
Carpet python nidovirus 1	TGS	6/15 (40%)	4/5 (80%)	2/7 (29%)	0/3 (0%)
Serpentovirus	TGS	1/15 (7%)	0/5 (0%)	1/7 (14%)	0/3 (0%)
Serpentovirus (PCR1, PCR2)	PCR	9/15 (60%)	4/5 (80%)	2/7 (29%)	3/3 (100%)
**Bacteria**					
*Mycoplasma agassizii* like (PCR1, PCR2)	PCR	15/15 (100%)	5/5 (100%)	7/7 (100%)	3/3 (100%)
*M. [Mycoplasmopsis] iguanae*	TGS	1/15 (7%)	1/5 (20%)	0/7 (0%)	0/3 (0%)
*M. [Mycoplasmoides] fastidiosum*	TGS	7/15 (47%)	2/5 (40%)	2/7 (29%)	3/3 (100%)
*M. [Mycoplasmopsis] agassizii*	TGS	6/15 (40%)	3/5 (60%)	3/7 (43%)	0/3 (0%)
*M. [Mycoplasmopsis] pulmonis*	TGS	6/15 (40%)	0/5 (0%)	3/7 (43%)	3/3 (100%)
*Mycoplasmopsis* sp.	TGS	2/15 (13%)	1/5 (20%)	1/7 (14%)	0/3 (0%)
*M. testudineum [Mycoplasmopsis testudinea]*	TGS	5/15 (33%)	3/5 (60%)	2/7 (29%)	0/3 (0%)
*Mesomycoplasma* sp.	TGS	1/15 (7%)	0/5 (0%)	1/7 (14%)	0/3 (0%)
*Chlamydia* sp.	PCR	2/15 (13%)	0/5 (0%)	0/7 (0%)	2/3 (67%)
*Chlamydia* sp.	TGS	1/15 (7%)	0/5 (0%)	0/7 (0%)	1/3 (33%)
*Bacteroides fragilis*	TGS	1/15 (7%)	0/5 (0%)	0/7 (0%)	1/3 (33%)
*Brucella intermedia* comb. nov basionym: *Ochrobactrum intermedium*	MALDI-TOF **	7/11 (64%)	4/5 (80%)	2/7 (29%)	1/3 (33%)
*Chryseobacterium* sp.	TGS	5/15 (33%)	2/5 (40%)	1/7 (14%)	2/3 (67%)
*Citrobacter* sp.	TGS	1/15 (7%)	0/5 (0%)	1/7 (14%)	0/3 (0%)
*Elizabethkingia* sp.	TGS	7/15 (47%)	3/5 (60%)	4/7 (57%)	0/3 (0%)
*Escherichia* sp.	TGS	5/15 (33%)	1/5 (20%)	4/7 (57%)	0/3 (0%)
*Flavobacterium* sp.	TGS	2/15 (13%)	1/5 (20%)	1/7 (14%)	0/3 (0%)
*Lysobacter pythonis*	TGS	2/15 (13%)	1/5 (20%)	0/7 (0%)	1/3 (33%)
*Paracoccus* sp.	TGS	2/15 (13%)	0/5 (0%)	0/7 (0%)	2/3 (67%)
*Providencia rettgeri*	TGS	1/15 (7%)	0/5 (0%)	0/7 (0%)	1/3 (33%)
*Pseudomonas* sp.	TGS	3/15 (20%)	0/5 (0%)	3/7 (43%)	0/3 (0%)

* Three Dumeril’s boas that were kept in the same terrarium. ** MALDI-TOF results refer to isolates obtained from mycoplasma cultivation attempts.

## Data Availability

The original contributions presented in the study are included in the article, further inquiries can be directed to the corresponding authors.

## Supplementary Materials

The following supporting information can be downloaded at: https://www.mdpi.com/article/10.3390/ani15152187/s1, Table S1: A summary of the examined snakes (clinically healthy), including the pathogens detected, categorized by diagnostic method and accompanied by the respective clinical findings. Table S2: A summary of the examined snakes with clinical signs including the pathogens detected, categorized by diagnostic method and accompanied by the respective clinical findings; Table S3: Sequence analysis results obtained using GenBank’s BLASTn and the leBIBI QBPP tool, including correlation of the identified mycoplasma sequences with previously described mycoplasma clades; Table S4: Overview of BLAST hits for detected serpentoviruses using TGS. Refs [109,110] are cited in supplementary materials.

## Abbreviations

The following abbreviations are used in this manuscript:

PCR	Polymerase chain reaction
TGS	Third-generation sequencing
ICTV	International Committee on Taxonomy of Viruses
BPNV-1	Ball Python Nidovirus 1
URT	Upper respiratory tract
LPSN	list of prokaryotic names with standing in nomenclature
MBA	Mycoplasmology, bacteriology, and antimicrobial resistance unit
BLAST	Basic Local Alignment Search Tool
MALDI-TOF	Matrix-assisted laser desorption/ionization-time of flight mass spectrometry
DNA	Deoxyribonucleic acid

## References

[1] Schmidt V., Marschang R.E., Abbas M.D., Ball I., Szabo I., Helmuth R., Plenz B., Spergser J., Pees M. (2013). Detection of pathogens in Boidae and Pythonidae with and without respiratory disease. Vet. Rec..

[2] Hoon-Hanks L.L., Ossiboff R.J., Bartolini P., Fogelson S.B., Perry S.M., Stöhr A.C., Cross S.T., Wellehan J.F.X., Jacobson E.R., Dubovi E.J. (2019). Longitudinal and Cross-Sectional Sampling of Serpentovirus (Nidovirus) Infection in Captive Snakes Reveals High Prevalence, Persistent Infection, and Increased Mortality in Pythons and Divergent Serpentovirus Infection in Boas and Colubrids. Front. Vet. Sci..

[3] Comolli J.R., Divers S.J. (2021). Respiratory Diseases of Snakes. Vet. Clin. Exot. Anim..

[4] Marschang R.E., Salzmann E., Pees M. (2021). Diagnostics of Infectious Respiratory Pathogens in Reptiles. Vet. Clin. N. Am. Exot. Anim. Pract..

[5] Hilf M., Wagner R.A., Yu V.L. (1990). A Prospective Study of Upper Airway Flora in Healthy Boid Snakes and Snakes with Pneumonia. J. Zoo Wildl. Med..

[6] Orós J., Rodríguez J.L., Herráez P., Santana P., Fernández A. (1996). Respiratory and digestive lesions caused by *Salmonella arizonae* in two snakes. J. Comp. Pathol..

[7] Govendan P.N., Purbantoro S., Erika E., Rumbay Y., Rompis A. (2022). Clinical Findings and Bacterial Identification in Eight Pythons with Respiratory Disorders in Bali. J. Vet..

[8] Lamirande E.W., Nichols D.K., Owens J.W., Gaskin J.M., Jacobson E.R. (1999). Isolation and experimental transmission of a reovirus pathogenic in ratsnakes (*Elaphe* species). Virus Res..

[9] Hyndman T.H., Marschang R.E., Wellehan J.F., Nicholls P.K. (2012). Isolation and molecular identification of Sunshine virus, a novel paramyxovirus found in Australian snakes. Infect. Genet. Evol..

[10] Hyndman T.H., Shilton C.M., Marschang R.E. (2013). Paramyxoviruses in reptiles: A review. Vet. Microbiol..

[11] Crossland N.A., DiGeronimo P.M., Sokolova Y., Childress A.L., Wellehan J.F.X., Nevarez J., Paulsen D. (2018). Pneumonia in a Captive Central Bearded Dragon with Concurrent Detection of Helodermatid Adenovirus 2 and a Novel Mycoplasma Species. Vet. Pathol..

[12] Hoon-Hanks L.L., Layton M.L., Ossiboff R.J., Parker J.S.L., Dubovi E.J., Stenglein M.D. (2018). Respiratory disease in ball pythons (*Python regius*) experimentally infected with ball python nidovirus. Virology.

[13] Gorbalenya A.E., Enjuanes L., Ziebuhr J., Snijder E.J. (2006). *Nidovirales*: Evolving the largest RNA virus genome. Virus Res..

[14] Walker P.J., Siddell S.G., Lefkowitz E.J., Mushegian A.R., Adriaenssens E.M., Alfenas-Zerbini P., Dempsey D.M., Dutilh B.E., García M.L., Curtis Hendrickson R. (2022). Recent changes to virus taxonomy ratified by the International Committee on Taxonomy of Viruses (2022). Arch. Virol..

[15] Zhu N., Zhang D., Wang W., Li X., Yang B., Song J., Zhao X., Huang B., Shi W., Lu R. (2020). A Novel Coronavirus from Patients with Pneumonia in China, 2019. N. Engl. J. Med..

[16] Parrish K., Kirkland P.D., Skerratt L.F., Ariel E. (2021). Nidoviruses in Reptiles: A Review. Front. Vet. Sci..

[17] Boon A., Iredale M., Tillis S., Ossiboff R. (2023). Ophidian Serpentoviruses: A Review and Perspective. J. Herpetol. Med. Surg..

[18] Tillis S.B., Josimovich J.M., Miller M.A., Hoon-Hanks L.L., Hartmann A.M., Claunch N.M., Iredale M.E., Logan T.D., Yackel Adams A.A., Bartoszek I.A. (2022). Divergent Serpentoviruses in Free-Ranging Invasive Pythons and Native Colubrids in Southern Florida, United States. Viruses.

[19] Bodewes R., Lempp C., Schürch A.C., Habierski A., Hahn K., Lamers M., von Dörnberg K., Wohlsein P., Drexler J.F., Haagmans B.L. (2014). Novel divergent nidovirus in a python with pneumonia. J. Gen. Virol..

[20] Stenglein M.D., Jacobson E.R., Wozniak E.J., Wellehan J.F., Kincaid A., Gordon M., Porter B.F., Baumgartner W., Stahl S., Kelley K. (2014). Ball python nidovirus: A candidate etiologic agent for severe respiratory disease in *Python regius*. mBio.

[21] Uccellini L., Ossiboff R.J., de Matos R.E.C., Morrisey J.K., Petrosov A., Navarrete-Macias I., Jain K., Hicks A.L., Buckles E.L., Tokarz R. (2014). Identification of a novel nidovirus in an outbreak of fatal respiratory disease in ball pythons (*Python regius*). Virol. J..

[22] O’Dea M., Jackson B., Jackson C., Xavier P., Warren K. (2016). Discovery and Partial Genomic Characterisation of a Novel Nidovirus Associated with Respiratory Disease in Wild Shingleback Lizards (*Tiliqua rugosa*). PLoS ONE.

[23] Zhang J., Finlaison D.S., Frost M.J., Gestier S., Gu X., Hall J., Jenkins C., Parrish K., Read A.J., Srivastava M. (2018). Identification of a novel nidovirus as a potential cause of large scale mortalities in the endangered Bellinger River snapping turtle (*Myuchelys georgesi*). PLoS ONE.

[24] Hoon-Hanks L.L., Stöhr A.C., Anderson A.J., Evans D.E., Nevarez J.G., Díaz R.E., Rodgers C.P., Cross S.T., Steiner H.R., Parker R.R. (2020). Serpentovirus (Nidovirus) and Orthoreovirus Coinfection in Captive Veiled Chameleons (*Chamaeleo calyptratus*) with Respiratory Disease. Viruses.

[25] Blahak S., Jenckel M., Höper D., Beer M., Hoffmann B., Schlottau K. (2020). Investigations into the presence of nidoviruses in pythons. Virol. J..

[26] Dervas E., Hepojoki J., Laimbacher A., Romero-Palomo F., Jelinek C., Keller S., Smura T., Hepojoki S., Kipar A., Hetzel U. (2017). Nidovirus-Associated Proliferative Pneumonia in the Green Tree Python (*Morelia viridis*). J. Virol..

[27] Dervas E., Hepojoki J., Smura T., Prähauser B., Windbichler K., Blümich S., Ramis A., Hetzel U., Kipar A. (2020). Serpentoviruses: More than Respiratory Pathogens. J. Virol..

[28] Brown M.B., Brown D.R., Klein P.A., McLaughlin G.S., Schumacher I.M., Jacobson E.R., Adams H.P., Tully J.G. (2001). *Mycoplasma agassizii* sp. nov., isolated from the upper respiratory tract of the desert tortoise (*Gopherus agassizii*) and the gopher tortoise (*Gopherus polyphemus*). Int. J. Syst. Evol. Microbiol..

[29] Brown D.R., Merritt J.L., Jacobson E.R., Klein P.A., Tully J.G., Brown M.B. (2004). *Mycoplasma testudineum* sp. nov., from a desert tortoise (*Gopherus agassizii*) with upper respiratory tract disease. Int. J. Syst. Evol. Microbiol..

[30] Jacobson E.R., Brown M.B., Wendland L.D., Brown D.R., Klein P.A., Christopher M.M., Berry K.H. (2014). Mycoplasmosis and upper respiratory tract disease of tortoises: A review and update. Vet. J..

[31] Penner J.D., Jacobson E.R., Brown D.R., Adams H.P., Besch-Williford C.L. (1997). A novel *Mycoplasma* sp. associated with proliferative tracheitis and pneumonia in a Burmese python (*Python molurus bivittatus*). J. Comp. Pathol..

[32] Marschang R., Heckers K., Dietz J., Kolesnik E. (2016). Detection of a mycoplasma in a python (*Morelia spilota*) with stomatitis. J. Herpetol. Med. Surg..

[33] Magalhães B., Machado L., Figueira A., Dias T., Feijó T., Barreto M., Tuffanelli G., Cunha N., Nascimento E., Pereira V. (2021). *Mycoplasma* spp. in captive snakes (*Boa constrictor* and *Bothrops atrox*) from Brazil. Ciênc. Rural.

[34] Faulhaber M.M., Tardy F., Saul F., Müller E., Pees M., Marschang R.E. (2025). Detection of *Mycoplasma* spp. from snakes from five different families. BMC Vet. Res..

[35] Gupta R.S., Sawnani S., Adeolu M., Alnajar S., Oren A. (2018). Correction to: Phylogenetic framework for the phylum Tenericutes based on genome sequence data: Proposal for the creation of a new order *Mycoplasmoidales* ord. nov., containing two new families *Mycoplasmoidaceae* fam. nov. and *Metamycoplasmataceae* fam. nov. harbouring *Eperythrozoon*, *Ureaplasma* and five novel genera. Antonie Van Leeuwenhoek.

[36] Gupta R.S., Oren A. (2020). Necessity and rationale for the proposed name changes in the classification of *Mollicutes* species. Reply to: ’Recommended rejection of the names *Malacoplasma* gen. nov., *Mesomycoplasma* gen. nov., *Metamycoplasma* gen. nov., *Metamycoplasmataceae* fam. nov., *Mycoplasmoidaceae* fam. nov., *Mycoplasmoidales* ord. nov., *Mycoplasmoides* gen. nov., *Mycoplasmopsis* gen. nov. [Gupta, Sawnani, Adeolu, Alnajar and Oren 2018] and all proposed species comb. nov. placed therein’, by M. Balish et al. (Int J Syst Evol Microbiol, 2019;69:3650–3653). Int. J. Syst. Evol. Microbiol..

[37] Sayers E.W., Bolton E.E., Brister J.R., Canese K., Chan J., Comeau D.C., Connor R., Funk K., Kelly C., Kim S. (2022). Database resources of the national center for biotechnology information. Nucleic Acids Res..

[38] Parte A.C., Sardà Carbasse J., Meier-Kolthoff J.P., Reimer L.C., Göker M. (2020). List of Prokaryotic names with Standing in Nomenclature (LPSN) moves to the DSMZ. Int. J. Syst. Evol. Microbiol..

[39] Yan X.-H., Pei S.-C., Yen H.-C., Blanchard A., Sirand-Pugnet P., Baby V., Gasparich G., Kuo C.-H. (2024). Delineating bacterial genera based on gene content analysis: A case study of the *Mycoplasmatales*-*Entomoplasmatales* clade within the class *Mollicutes*. Microb. Genom..

[40] Wellehan J.F., Johnson A.J., Harrach B., Benkö M., Pessier A.P., Johnson C.M., Garner M.M., Childress A., Jacobson E.R. (2004). Detection and analysis of six lizard adenoviruses by consensus primer PCR provides further evidence of a reptilian origin for the atadenoviruses. J. Virol..

[41] VanDevanter D., Warrener P., Bennett L., Schultz E.R., Coulter S., Garber R.L., Rose T. (1996). Detection and analysis of diverse herpesviral species by consensus primer PCR. J. Clin. Microbiol..

[42] Wellehan J., Johnson A., Latimer K., Whiteside D., Crawshaw G., Detrisac C., Terrell S., Heard D., Childress A., Jacobson E. (2005). Varanid herpesvirus 1: A novel herpesvirus associated with proliferative stomatitis in green tree monitors (*Varanus prasinus*). Vet. Microbiol..

[43] Catoi C., Gal A.F., Taulescu M.A., Palmieri C., Catoi A.F. (2014). Lethal herpesvirosis in 16 captive horned vipers (*Vipera ammodytes ammodytes*): Pathological and ultrastructural findings. J. Comp. Pathol..

[44] Hetterich J., Mirolo M., Kaiser F., Ludlow M., Reineking W., Zdora I., Hewicker-Trautwein M., Osterhaus A.D.M.E., Pees M. (2024). Concurrent Detection of a Papillomatous Lesion and Sequence Reads Corresponding to a Member of the Family Adintoviridae in a Bell’s Hinge-Back Tortoise (*Kinixys belliana*). Animals.

[45] Thiele T., Baggio F., Prähauser B., Ruiz Subira A., Michalopoulou E., Kipar A., Hetzel U., Hepojoki J. (2023). Reptarenavirus S Segment RNA Levels Correlate with the Presence of Inclusion Bodies and the Number of L Segments in Snakes with Reptarenavirus Infection-Lessons Learned from a Large Breeding Colony. Microbiol. Spectr..

[46] Argenta F.F., Hepojoki J., Smura T., Szirovicza L., Hammerschmitt M.E., Driemeier D., Kipar A., Hetzel U. (2020). Identification of Reptarenaviruses, Hartmaniviruses, and a Novel Chuvirus in Captive Native Brazilian Boa Constrictors with Boid Inclusion Body Disease. J. Virol..

[47] Houpikian P., Raoult D. (2002). Traditional and molecular techniques for the study of emerging bacterial diseases: One laboratory’s perspective. Emerg. Infect. Dis..

[48] Stenglein M.D., Sanders C., Kistler A.L., Ruby J.G., Franco J.Y., Reavill D.R., Dunker F., Derisi J.L. (2012). Identification, characterization, and in vitro culture of highly divergent arenaviruses from boa constrictors and annulated tree boas: Candidate etiological agents for snake inclusion body disease. mBio.

[49] Wellehan J.F., Childress A.L., Marschang R.E., Johnson A.J., Lamirande E.W., Roberts J.F., Vickers M.L., Gaskin J.M., Jacobson E.R. (2009). Consensus nested PCR amplification and sequencing of diverse reptilian, avian, and mammalian orthoreoviruses. Vet. Microbiol..

[50] Sachse K., Hotzel H., Slickers P., Ehricht R. (2006). The use of DNA microarray technology for detection and genetic characterisation of Chlamydiae. Dev. Biol..

[51] Geneious Prime 2025.0.3. http://www.geneious.com/.

[52] National Center for Biotechnology Information (NCBI) [Internet] (1988). Bethesda (MD): National Library of Medicine (US), National Center for Biotechnology Information. https://www.ncbi.nlm.nih.gov/.

[53] Altschul S.F., Gish W., Miller W., Myers E.W., Lipman D.J. (1990). Basic local alignment search tool. J. Mol. Biol..

[54] Flandrois J.P., Perrière G., Gouy M. (2015). leBIBIQBPP: A set of databases and a webtool for automatic phylogenetic analysis of prokaryotic sequences. BMC Bioinform..

[55] Theuns S., Vanmechelen B., Bernaert Q., Deboutte W., Vandenhole M., Beller L., Matthijnssens J., Maes P., Nauwynck H.J. (2018). Nanopore sequencing as a revolutionary diagnostic tool for porcine viral enteric disease complexes identifies porcine kobuvirus as an important enteric virus. Sci. Rep..

[56] Vereecke N., Zwickl S., Gumbert S., Graaf A., Harder T., Ritzmann M., Lillie-Jaschniski K., Theuns S., Stadler J. (2023). Viral and Bacterial Profiles in Endemic Influenza A Virus Infected Swine Herds Using Nanopore Metagenomic Sequencing on Tracheobronchial Swabs. Microbiol. Spectr..

[57] Bokma J., Vereecke N., Pas M.L., Chantillon L., Vahl M., Weesendorp E., Deurenberg R.H., Nauwynck H., Haesebrouck F., Theuns S. (2021). Evaluation of Nanopore Sequencing as a Diagnostic Tool for the Rapid Identification of *Mycoplasma bovis* from Individual and Pooled Respiratory Tract Samples. J. Clin. Microbiol..

[58] Van Herzele C., Coppens S., Vereecke N., Theuns S., de Graaf D.C., Nauwynck H. (2024). New insights into honey bee viral and bacterial seasonal infection patterns using third-generation nanopore sequencing on honey bee haemolymph. Vet. Res..

[59] Frey M.L., Hanson R.P., Andrson D.P. (1968). A medium for the isolation of avian mycoplasmas. Am. J. Vet. Res..

[60] Freundt E.A., Razin S., Tully J.G. (1983). C7-Culture Media for Classic Mycoplasmas. Methods in Mycoplasmology.

[61] Friis N.F. (1975). Some recommendations concerning primary isolation of *Mycoplasma suipneumoniae* and *Mycoplasma flocculare* a survey. Nord. Vet. Med..

[62] Cisneros-Tamayo M., Kempf I., Coton J., Michel V., Bougeard S., de Boisséson C., Lucas P., Bäyon-Auboyer M.H., Chiron G., Mindus C. (2020). Investigation on eggshell apex abnormality (EAA) syndrome in France: Isolation of *Mycoplasma synoviae* is frequently associated with *Mycoplasma pullorum*. BMC Vet. Res..

[63] Racz K., Salzmann E., Müller E., Marschang R.E. (2021). Detection of *Mycoplasma* and *Chlamydia* in Pythons With and Without Serpentovirus Infection. J. Zoo Wildl. Med..

[64] Hördt A., López M.G., Meier-Kolthoff J.P., Schleuning M., Weinhold L.M., Tindall B.J., Gronow S., Kyrpides N.C., Woyke T., Göker M. (2020). Analysis of 1,000+ Type-Strain Genomes Substantially Improves Taxonomic Classification of *Alphaproteobacteria*. Front. Microbiol..

[65] Pees M., Schmidt V., Marschang R.E., Heckers K.O., Krautwald-Junghanns M.E. (2010). Prevalence of viral infections in captive collections of boid snakes in Germany. Vet. Rec..

[66] Wellehan J.F.X., Divers S.J., Divers S.J., Stahl S.J. (2019). 29-Bacteriology. Mader’s Reptile and Amphibian Medicine and Surgery.

[67] Brown M., Schumacher I., Klein P., Harris K., Correll T., Jacobson E. (1994). *Mycoplasma agassizii* causes upper respiratory tract disease in the desert tortoise. Infect. Immun..

[68] Brown M., McLaughlin G., Klein P., Crenshaw B., Schumacher I., Brown D., Jacobson E. (1999). Upper respiratory tract disease in the gopher tortoise is caused by *Mycoplasma agassizii*. J. Clin. Microbiol..

[69] Jacobson E.R., Berry K.H. (2012). *Mycoplasma testudineum* in free-ranging desert tortoises, *Gopherus agassizii*. J. Wildl. Dis..

[70] Plenz B., Schmidt V., Grosse-Herrenthey A., Krüger M., Pees M. (2015). Characterisation of the aerobic bacterial flora of boid snakes: Application of MALDI-TOF mass spectrometry. Vet. Rec..

[71] Zancolli G., Mahsberg D., Sickel W., Keller A. (2015). Reptiles as Reservoirs of Bacterial Infections: Real Threat or Methodological Bias?. Microbial. Ecol..

[72] Blaylock R.S. (2001). Normal oral bacterial flora from some southern African snakes. Onderstepoort J. Vet. Res..

[73] Walker P.J., Siddell S.G., Lefkowitz E.J., Mushegian A.R., Adriaenssens E.M., Dempsey D.M., Dutilh B.E., Harrach B., Harrison R.L., Hendrickson R.C. (2020). Changes to virus taxonomy and the Statutes ratified by the International Committee on Taxonomy of Viruses (2020). Arch. Virol..

[74] Tillis S.B., Ossiboff R.J., Wellehan J.F.X. (2024). Serpentoviruses Exhibit Diverse Organization and ORF Composition with Evidence of Recombination. Viruses.

[75] Panda S.K., Padhi L., Sahoo G. (2018). Oral bacterial flora of Indian cobra (*Naja naja*) and their antibiotic susceptibilities. Heliyon.

[76] Busse H.J., Huptas C., Baumgardt S., Loncaric I., Spergser J., Scherer S., Wenning M., Kämpfer P. (2019). Proposal of *Lysobacter pythonis* sp. nov. isolated from royal pythons (*Python regius*). Syst. Appl. Microbiol..

[77] Artavia-León A., Romero-Guerrero A., Sancho-Blanco C., Rojas N., Umaña-Castro R. (2017). Diversity of Aerobic Bacteria Isolated from Oral and Cloacal Cavities from Free-Living Snakes Species in Costa Rica Rainforest. Int. Sch. Res. Not..

[78] Parrish K., Kirkland P., Horwood P., Chessman B., Ruming S., McGilvray G., Rose K., Hall J., Skerratt L. (2024). Delving into the Aftermath of a Disease-Associated Near-Extinction Event: A Five-Year Study of a Serpentovirus (Nidovirus) in a Critically Endangered Turtle Population. Viruses.

[79] Fonseca M., Moreira W.M.Q., da Cunha K., Ribeiro A., Almeida M. (2009). Oral microbiota of Brazilian captive snakes. J. Venom. Anim. Toxins Incl. Trop. Dis..

[80] Taylor-Brown A., Bachmann N.L., Borel N., Polkinghorne A. (2016). Culture-independent genomic characterisation of Candidatus *Chlamydia sanzinia*, a novel uncultivated bacterium infecting snakes. BMC Genom..

[81] Taylor-Brown A., Rüegg S., Polkinghorne A., Borel N. (2015). Characterisation of *Chlamydia pneumoniae* and other novel chlamydial infections in captive snakes. Vet. Microbiol..

[82] Myers G.S., Mathews S.A., Eppinger M., Mitchell C., O’Brien K.K., White O.R., Benahmed F., Brunham R.C., Read T.D., Ravel J. (2009). Evidence that human *Chlamydia pneumoniae* was zoonotically acquired. J. Bacteriol..

[83] Bodetti T.J., Jacobson E., Wan C., Hafner L., Pospischil A., Rose K., Timms P. (2002). Molecular evidence to support the expansion of the hostrange of *Chlamydophila pneumoniae* to include reptiles as well as humans, horses, koalas and amphibians. Syst. Appl. Microbiol..

[84] Jacobson E.R., Heard D., Andersen A. (2004). Identification of *Chlamydophila pneumoniae* in an emerald tree boa, *Corallus caninus*. J. Vet. Diagn. Invest..

[85] Soldati G., Lu Z.H., Vaughan L., Polkinghorne A., Zimmermann D.R., Huder J.B., Pospischil A. (2004). Detection of Mycobacteria and Chlamydiae in Granulomatous Inflammation of Reptiles: A Retrospective Study. Vet. Pathol..

[86] Staub E., Marti H., Biondi R., Levi A., Donati M., Leonard C.A., Ley S.D., Pillonel T., Greub G., Seth-Smith H.M.B. (2018). Novel *Chlamydia* species isolated from snakes are temperature-sensitive and exhibit decreased susceptibility to azithromycin. Sci. Rep..

[87] Jacobson E.R., Gaskin J.M., Mansell J. (1989). Chlamydial Infection in Puff Adders, *Bitis arietans*. J. Zoo Wildl. Med..

[88] Lock B., Heard D., Detrisac C., Jacobson E. (2003). An epizootic of chronic regurgitation associated with Chlamydophilosis in recently imported emerald tree boas (*Corallus caninus*). J. Zoo Wildl. Med..

[89] Jacobson E., Origgi F., Heard D., Detrisac C. (2002). Immunohistochemical Staining of Chlamydial Antigen in Emerald Tree Boas (*Corallus Caninus*). J. Vet. Diagn. Invest..

[90] Rüegg S.R., Regenscheit N., Origgi F.C., Kaiser C., Borel N. (2015). Detection of *Chlamydia pneumoniae* in a collection of captive snakes and response to treatment with marbofloxacin. Vet. J..

[91] Ryan M.P., Pembroke J.T. (2020). The Genus *Ochrobactrum* as Major Opportunistic Pathogens. Microorganisms.

[92] Wernick M., Novo Matos J., Ebling A., Kühn Campbell K., Ruetten M., Hilbe M., Howard J., Chang R., Prohaska S., Hatt J.-M. (2015). Valvulopathy consistent with endocarditis in an Argentine boa (*Boa constrictor occidentalis*). J. Zoo Wildl. Med..

[93] Velasco J., Romero C., López-Goñi I., Leiva J., Díaz R., Moriyón I. (1998). Evaluation of the relatedness of *Brucella* spp. and *Ochrobactrum anthropi* and description of *Ochrobactrum intermedium* sp. nov., a new species with a closer relationship to Brucella spp. Int. J. Syst. Bacteriol..

[94] Pasterny J., Skomorucha Ł., Stanicki K., Marschang R.E. (2021). Detection of Infectious Agents in Samples from Reptiles Presented at Veterinary Clinics in Poland. J. Herpetol. Med. Surg..

[95] Flanders A.J., Ossiboff R.J., Wellehan J.F.X., Alexander A.B., Fredholm D.V.E., Desiderio T.M., Stacy N.I. (2021). Presumptive heterophil extracellular traps recognized cytologically in nine reptile patients with inflammatory conditions. Vet. Q..

[96] Marschang R.E., Meddings J.I., Ariel E., Hurst C.J. (2021). Viruses of reptiles. Studies in Viral Ecology.

[97] Kischinovsky M., Raftery A., Sawmy S., Doneley D., Monks D., Johnson R., Carmel B. (2017). Husbandry and nutrition. Reptile Medicine and Surgery in Clinical Practice.

[98] Lazarkevich I., Engibarov S., Mitova S., Popova S., Vacheva E., Stanchev N., Eneva R., Gocheva Y., Lalovska I., Paunova-Krasteva T. (2024). Pathogenic Potential of Opportunistic Gram-Negative Bacteria Isolated from the Cloacal Microbiota of Free-Living Reptile Hosts Originating from Bulgaria. Life.

[99] Barazorda Romero S., Cizek A., Masarikova M., Knotek Z. (2015). Choanal and cloacal aerobic bacterial flora in captive green iguanas: A comparative analysis. Acta. Vet. Brno..

[100] Stahl S.J. How I approach snake respiratory disease: The five-minute consult. Proceedings of the NAVC Conference.

[101] Knotek Z., Divers S.J., Divers S.J., Stahl S.J. (2019). 76-Pulmonology. Mader’s Reptile and Amphibian Medicine and Surgery.

[102] Isaza R., Jacobson E.R. (2013). Antimicrobial Drug Use in Reptiles. Antimicrobial Therapy in Veterinary Medicine.

[103] Lawrence K., Muggleton P.W., Needham J.R. (1984). Preliminary study on the use of ceftazidime, a broad spectrum cephalosporin antibiotic, in snakes. Res. Vet. Sci..

[104] Sonntag F.D., Rüschoff B., Troll C., Heckers K.O., Marschang R.E. (2021). Bacteria Associated with Clinically Suspected Respiratory Disease in Snakes and Effective Antimicrobial Treatment Options. J. Herpetol. Med. Surg..

[105] Damerum A., Malka S., Lofgren N., Vecere G., Krumbeck J.A. (2023). Next-generation DNA sequencing offers diagnostic advantages over traditional culture testing. Am. J. Vet. Res..

[106] Dipineto L., Russo T.P., Calabria M., De Rosa L., Capasso M., Menna L.F., Borrelli L., Fioretti A. (2014). Oral flora of Python regius kept as pets. Lett. Appl. Microbiol..

[107] Starck J.M., Weimer I., Aupperle H., Müller K., Marschang R.E., Kiefer I., Pees M. (2015). Morphological Pulmonary Diffusion Capacity for Oxygen of Burmese Pythons (*Python molurus*): A Comparison of Animals in Healthy Condition and with Different Pulmonary Infections. J. Comp. Pathol..

[108] Marschang R.E., Kolesnik E. (2017). Detection of nidoviruses in live pythons and boas. Tierarztl Prax. Ausg. Kleintiere Heimtiere.

[109] Brown D.R., Crenshaw B.C., McLaughlin G.S., Schumacher I.M., McKenna C.E., Klein P.A., Jacobson E.R., Brown M.B. (1995). Taxonomic analysis of the tortoise mycoplasmas Mycoplasma agassizii and Mycoplasma testudinis by 16S rRNA gene sequence comparison. Int. J. Syst. Bacteriol..

[110] van Kuppeveld F.J., van der Logt J.T., Angulo A.F., van Zoest M.J., Quint W.G., Niesters H.G., Galama J.M., Melchers W.J. (1992). Genus- and species-specific identification of mycoplasmas by 16S rRNA amplification. Appl. Environ. Microbiol..

