# Supplementary material for: Identifying Infectious Agents in Snakes (Boidae and Pythonidae) with and Without Respiratory Disease
Source: Animals (Basel). 2025 Jul 25;15(15):2187. doi: 10.3390/ani15152187 (PMC12345485; doi:10.3390/ani15152187)
Supplement: Supplementary file 1 [file animals-15-02187-s001.zip › animals-3736514-supplementary.pdf]

Supplementary Material

Supplementary Table S1. A summary of the examined snakes (clinically healthy), including the pathogens detected, categorized by diagnostic method and accompanied by the respective clinical findings

**Species**	*Python regius* (Ball Python)	*Python regius* (Ball Python)	*Python regius* (Ball Python)	*Python regius* (Ball Python)	*Python regius* (Ball Python)	*Python curtus* (Sumatra Python)	*Malayopython reticulatus* (Reticulated Python)	*Acrantophis dumerili* (Dumeril's Boa)	*Acrantophis dumerili* (Dumeril's Boa)
**Sample type**	Swab	Swab	Swab	Swab	Swab	Swab	Swab	Swab	Swab
**Sample ID**	**K09608**	**K09602**	**K09609**	**K02321**	**K02325**	**K02330**	**K02324**	**K09605**	**K09603**
**Results TGS (very low, low, medium, high, very high)**									
**Viruses**									
Bellinger River related virus (potential novel)								Very High	Very High
Carpet python nidovirus 1	Medium		Medium						
Serpentovirus						Low			
**Bacteria**									
Mesomycoplasma sp.				Low					
M. testudineum [Mycoplasmopsis testudinea]	Very low						High		
M. [Mycoplasmoides] fastidiosum		Very low				Low		Low	Very low
M. [Mycoplasmopsis] iguanae									
Mycoplasmopsis sp.	Very low								
M. [Mycoplasmopsis] pulmonis			Low		Low	Low		Low	Very low
M. [Mycoplasmopsis] agassizii			Very low		Low		Medium		
Bacteroides fragilis									
Chryseobacterium sp.	Very low							Very low	

Elizabethkingia sp.				High	High	Medium	High		
Flavobacterium sp.	Very low								
Citrobacter sp.	Very low								
Escherichia sp.				Medium	Low	High	Medium		
Lysobacter pythonis								Very low	
Chlamydia sp.								Very low	
Pseudomonas sp.				Low	Low		Low		
**Results PCR (positive, negative)**									
**Viruses**									
Serpentovirus PCR1 [25]	positive	negative	positive	negative	negative	negative	negative	positive	positive
Serpentovirus PCR2 [20]	positive	negative	positive	negative	negative	negative	negative	positive	positive
Adenovirus	negative	negative	negative	negative	negative	negative	negative	negative	negative
Arenavirus	negative	negative	negative	negative	negative	negative	negative	negative	negative
Ferlavirus	negative	negative	negative	negative	negative	negative	negative	negative	negative
Reovirus	negative	negative	negative	negative	negative	negative	negative	negative	negative
**Bacteria**									
Mycoplasma spp. PCR1 [109]	positive	positive	positive	positive	positive	positive	positive	positive	positive
Mycoplasma spp. PCR2 [110]	positive	positive	positive	positive	positive	positive	positive	positive	positive
Chlamydia sp.	negative	negative	negative	negative	negative	negative	negative	slightly positive	slightly positive
**Mycoplasma isolation**									
Mycoplasma spp.- like colonies	Not identified	Not identified	Identified	n.d.*	n.d.*	n.d.*	n.d.*	Identified	Not identified
**MALDI-TOF results**									

**Pathogens identified**									
Brucella intermedia comb. nov.	Identified	Not identified	Identified	n.d.*	n.d.*	n.d.*	n.d.*	Not identified	Not identified
**Clinical signs**									
**Clinical signs observed**	**Healthy**	**Healthy**	**Healthy**	**Healthy**	**Healthy**	**Healthy**	**Healthy**	**Healthy**	**Healthy**
Deceased animals	exitus 23.10.2023							exitus 31.03.2025	exitus 12.2024

*n.d. = not done

Supplementary Table S2. A summary of the examined snakes with clinical signs including the pathogens detected, categorized by diagnostic method and accompanied by the respective clinical findings

**Species**	*Acrantophis dumerili* (Dumeril's Boa)	*Python anchietae* (Angolan Python)	*Python regius* (Ball Python)	*Python regius* (Ball Python)	*Python regius* (Ball Python)	*Python regius* (Ball Python)
**Sample type**	Swab	Swab	Swab	Swab	Swab	Swab
**Sample ID**	**K09604**	**K09606**	**K09601**	**K09607**	**K09610**	**K09611**
**Results TGS (very low, low, medium, high, very high)**						
**Viruses**						
Bellinger River related virus (potential novel)	High					
Carpet python nidovirus 1		Very low		Low	Very low	Medium
Serpentovirus						
**Bacteria**						
Mesomycoplasma sp.						
M. testudineum [Mycoplasmopsis testudinea]			Very low		Very low	Very low
M. [Mycoplasmoides] fastidiosum	Very low		Very low			Very low
M. [Mycoplasmopsis] iguanae		Very low				
Mycoplasmopsis sp.			Very low			
M. [Mycoplasmopsis] pulmonis	Very low					
M. [Mycoplasmopsis] agassizii		Very low			Very low	Very low
Bacteroides fragilis	Very low					
Chryseobacterium sp.	Very low	Very low	Very low			
Elizabethkingia sp.		Very low		Very low		Low
Flavobacterium sp.						Very low

Citrobacter sp.						
Escherichia sp.						Very low
Lysobacter pythonis						Very low
Chlamydia sp.						
Pseudomonas sp.						
**Results PCR (positive, negative)**						
**Viruses**						
Serpentovirus PCR1 [25]	positive	positive	negative	positive	positive	positive
Serpentovirus PCR2 [20]	positive	positive	negative	positive	positive	positive
Adenovirus	negative	negative	negative	negative	negative	negative
Arenavirus	negative	negative	negative	negative	negative	negative
Ferlavirus	negative	negative	negative	negative	negative	negative
Reovirus	negative	negative	negative	negative	negative	negative
**Bacteria**						
Mycoplasma spp. PCR1 ([109])	positive	positive	positive	positive	positive	positive
Mycoplasma spp. PCR2 ([110])	positive	positive	positive	positive	positive	positive
Chlamydia sp.	negative	negative	negative	negative	negative	negative
**Mycoplasma isolation**						
Mycoplasma spp.- like colonies	Not identified YES	Identified YES	Identified NO	Not identified NO	Not identified YES	Identified
**MALDI-TOF results**						
**Pathogens identified**						
Brucella intermedia comb. nov.	Identified YES	Identified YES	Identified NO	Identified NO	Not identified YES	Identified

**Clinical signs**						
**Clinical signs observed**	**Diseased**	**Diseased**	**Diseased**	**Diseased**	**Diseased**	**Diseased**
Nares	nasal discharge	None	None	None	None	None
Oral cavity	mucous fluid, hyperemia of the mucous membranes	mucous fluid	mucous fluid hyperemia of the mucous membranes, wheezing	mucous fluid, cream-colored coating	mucous fluid, wheezing	mucous fluid
Deceased animals	exitus 24.03-25	exitus 25.09.2023				

Supplementary Table S3. Sequence analysis results obtained using GenBank's BLASTn and the leBIBI QBPP tool, including correlation of the identified Mycoplasma sequences with previously described Mycoplasma clades.

**Species**	**Sample ID**	**BLASTn first hit *Mycoplasma* spp. PCR1 products (according to [34])**	**leBIBIQBPP first hit of *Mycoplasma* spp. PCR1 products and assignment to clades (according to [34])**	**BLASTn first hit of *Mycoplasma* spp. PCR2 products and assignment to clades (according to [34])**
Python regius (Ball Python)	**K09601**	insufficient quality for an evaluation	insufficient quality for an evaluation	MZ686534.1 [63] Cluster A [34]
Python regius (Ball Python)	**K09602**	MZ686543.1 [63] Cluster A [34]	U09786 ([109]) AY366210 [29] Cluster A/B [34]	MZ686534.1 [63] Cluster A [34]
Python regius (Ball Python)	**K09607**	MZ686543.1 [63] Cluster A [34]	U09786 ([109]) AY366210 [29] Cluster A/B [34]	insufficient quality for an evaluation
Python regius (Ball Python)	**K09608**	MZ686543.1 [63] Cluster A [34]	U09786 ([109]) AY366210 [29] Cluster A/B [34]	insufficient quality for an evaluation
Python regius (Ball Python)	**K09609**	MZ686541.1 [63] Cluster A [34]	AY366210 [29] Cluster A/B [34]	insufficient quality for an evaluation
Python regius (Ball Python)	**K09610**	MZ686543.1 [63] Cluster A [34]	U09786 ([109]) Cluster A/B [34]	insufficient quality for an evaluation
Python regius (Ball Python)	**K09611**	MZ686543.1 [63] Cluster A [34]	U09786 ([109]) AY366210 [29] Cluster A/B [34]	insufficient quality for an evaluation
Python regius (Ball Python)	**K02321**	insufficient quality for an evaluation	insufficient quality for an evaluation	MZ686534.1 [63] Cluster A [34]
Python regius (Ball Python)	**K02325**	MZ686543.1 [63] Cluster A [34]	AY366210 [29] U09786 ([109]) Cluster A/B [34]	MZ686534.1 [63] Cluster A [34]
Malayopython reticulatus (Reticulated Python)	**K02324**	MZ686543.1 [63] Cluster A [34]	AY366210 [29] U09786 ([109]) Cluster A/B [34]	MZ686534.1 [63] Cluster A [34]
Python curtus (Sumatra Python)	**K02330**	MZ686543.1 [63] Cluster A [34]	AY366210 [29] U09786 ([109]) Cluster A/B [34]	MZ686534.1 [63] Cluster A [34]
Python anchietae (Angolan Python)	**K09606**	MZ686543.1 [63] Cluster A [34]	AY366210 [29] U09786 ([109]) Cluster A/B [34]	MZ686534.1 [63] Cluster A [34]

Acrantophis dumerili (Dumeril's Boa)	**K09603**	KU862617.1 [32] Cluster A [34]	AY366210 [29] Cluster A/B [34]	MZ686541.1 [63] Cluster A [34]
Acrantophis dumerili (Dumeril's Boa)	**K09604**	insufficient quality for an evaluation	insufficient quality for an evaluation	MZ686541.1 [63] Cluster A [34]
Acrantophis dumerili (Dumeril's Boa)	**K09605**	KU862617.1 [32] Cluster A [34]	AY366210 [29] Cluster A/B [34]	MZ686534.1 [63] Cluster A [34]

Supplementary Table S4. Overview of BLAST hits for detected serpentoviruses using TGS

**Snake ID**	**Taxon**	**Contig length**	**GenBank accession number(s)**	**Top hit**	**Query cover**	**% identity**
**K02330**	Serpentovirus	9350bp	PV975819	OR131603.1, Serpentovirinae sp. isolate 5422E1_Blood_1	98%	84.53%
**K09603**	Bellinger River related virus	25862bp	PV975820	NC_046956, Bellinger River virus isolate J248, complete genome	64%	72.58%
**K09604**	Bellinger River related virus	31156bp	PV975821	NC_046956, Bellinger River virus isolate J248, complete genome	52%	72.29%
**K09605**	Bellinger River related virus	25281bp	PV975822	NC_046956, Bellinger River virus isolate J248, complete genome	64%	72.53%
**K09606**	Carpet python nidovirus 1	N/A*	N/A	MK722366.1, Carpet python nidovirus 1 strain F17-605-7, complete genome	100%	91.94%
**K09607**	Carpet python nidovirus 1	10936bp	PV975823	MK722366.1 = Carpet python nidovirus 1 strain F17-605-7, complete genome	100%	97.29%
**K09608**	Carpet python nidovirus 1	13838bp	PV975824-PV975828	MK722366.1 = Carpet python nidovirus 1 strain F17-605-7, complete genome	94%	97.44%
**K09609**	Carpet python nidovirus 1	12008bp	PV975829-PV975833	MK722366.1 = Carpet python nidovirus 1 strain F17-605-7, complete genome	97%	97.31%
**K09610**	Carpet python nidovirus 1	19140bp	PV975834-PV975936	MK722366.1, Carpet python nidovirus 1 strain F17-605-7, complete genome	100%	96.76%
**K09611**	Carpet python nidovirus 1	14648bp	PV975837-PV975839	MK722366.1, Carpet python nidovirus 1 strain F17-605-7, complete genome	100%	96.77%

* Insufficient data for genome assembly
